# Computational methods for visualizing and measuring verapamil efficacy for cerebral vasospasm

**DOI:** 10.1038/s41598-020-75365-2

**Published:** 2020-11-02

**Authors:** Andrew Abumoussa, Alex Flores, James Ho, Marc Niethammer, Deanna Sasaki-Adams, Yueh Z. Lee

**Affiliations:** 1 Department of Neurosurgery, University of North Carolina, Physicians Office Building, 170 Manning Drive, Campus Box 7060, Chapel Hill, NC 27599 USA; 2 School of Medicine, University of North Carolina, Chapel Hill, NC 27514 USA; 3 Department of Radiology, University of North Carolina, Chapel Hill, NC 27514 USA; 4 Department of Computer Science, University of North Carolina, Chapel Hill, NC 27514 USA

**Keywords:** Neuro-vascular interactions, Stroke, Stroke

## Abstract

Cerebral vasospasm is a dreaded sequelae of aneurysmal subarachnoid hemorrhage (aSAH), requiring timely intervention with therapeutic goals of improving brain perfusion. There are currently no standardized real-time, objective assessments of the interventional procedures performed to treat vasospasm. Here we describe real-time techniques to quantify cerebral perfusion during interventional cerebral angiography. We retrospectively analyzed 39 consecutive cases performed to treat clinical vasospasm and quantified the changes in perfusion metrics between pre- and post- verapamil administrations. With Digital Subtraction Angiography (DSA) perfusion analysis, we are able to identify hypoperfused territories and quantify the exact changes in cerebral perfusion for each individual case and vascular territory. We demonstrate that perfusion analysis for DSA can be performed in real time. This provides clinicians with a colorized map which directly visualizes hypoperfused tissue, combined with associated perfusion statistics. Quantitative thresholds and analysis based on DSA perfusion may assist with real-time dosage estimation and help predict response to treatment, however future prospective analysis is required for validation.

## Introduction

Cerebral vasospasm is one of the dreaded complications of aneurysmal subarachnoid hemorrhage (aSAH) and is the cause of significant associated morbidity and mortality^1,2^. Vasospasm following aSAHs is thought to approach 60%^3^. When vasospasm is suspected, a timely multi-modal approach is utilized to both diagnose and treat this complication^4–9^. With a confirmed, or even suspected, diagnosis, goals of therapy immediately become to improve brain perfusion in order to minimize the risk for delayed cerebral ischemia which would result in permanent neurological deficit. Current treatments involve intravascular volume optimization and interventional procedures such as cerebral angioplasty or selective intra-arterial (IA) vasodilation performed under Digital Subtraction Angiography (DSA)^10,11^.

Though IA vasodilators have been shown to be effective and have become standard therapy in many institutions there remains no standardized method to objectively measure the efficacy of this invasive therapy^12,13^. During the treatment, efficacy is assessed by a subjective assessment of the change in vascular lumen size. This proxy can be misleading as radiographic vasospasm does not always correlate to clinical vasospasm or outcome, especially when cerebral perfusion is was not improved despite the IA therapy. As such, a more direct and rigorous method for measuring perfusion is needed.

Perfusion angiography, or quantitative analysis and visualization of blood flow parameters from DSA, has recently been presented as a novel imaging tool to assess blood physiology in the setting of vascular pathology^12^. Though results have been promising, data on the utility of perfusion angiography for vasospasm remains limited, particularly with use of IA verapamil, our institution’s first line IA agent^14,15^. With this work we present real-time techniques to quantify cerebral perfusion using DSA in order to quantify the efficacy of IA verapamil therapy.

## Methods

### Patients

A retrospective study was approved by our institution’s IRB committee and all of this research was performed in accordance with our institution’s guidelines and regulations, with a waiver of consent granted. The imaging dataset was collected at a single institution and included only cerebral angiography of patients undergoing DSA for clinical vasospasm with corresponding radiographic vasospasm who then underwent endovascular treatment during their hospitalization for aSAH. We retrospectively identified 39 patients during the time period of 2000 to 2019 who presented to our institution with aSAH, subsequently demonstrated signs and symptoms of cerebral vasospasm and then required invasive treatment via IA treatment. All patients were deemed appropriate candidates for IA therapy by our neurointensive care, neurosurgical, and neurointerventional providers.

Inclusion criteria for this study were: (1) patients admitted with the primary diagnosis of aSAH, (2) a decline in the neurological examination was identified during the vasospasm window, (3) the patient was eligible for and received IA verapamil, and (4) both pre-verapamil and post-verapamil administration angiograms were performed on the same vascular territory during the therapeutic intervention. Our exclusion criteria for not performing a quantitative analysis of a DSA study were (1) significant patient motion during the angiography of the verapamil injection, (2) pre/post studies were not of the same view, (3) the pre/post studies utilized different magnifications or (4) the angiographic study did not capture the entire arterial, capillary and venous phases.

Thirty-nine patients underwent IA verapamil therapy for vasospasm during the study period. Collectively, there were 52 therapeutic interventions with a total of 95 administrations of verapamil. Females suffered a disproportionate burden of disease with 32 cases (82%) as compared to 7 male patients (18%). The average age of patients experiencing vasospasm for this population was 48.6 ± 16 years for males (one male patient was 13 years old, removing this outlier the average age is 54.5 ± 3.7 years) and 51.5 ± 9.2 years for females (Table 1).

### Angiographic acquisition

DSA examinations were performed by two interventional neuroradiologists and one board certified endovascular neurosurgeon. Images were obtained using a biplane angiography suite (Artis Zeego, Siemens AG, Erlangen, Germany). According to our institution’s standard protocol, the patients were placed in a supine position on the angiography table, both groins were prepped and draped in the usual sterile fashion, and vascular access was obtained using the Seldinger technique. A 5-French, 10-cm sheath (Pinnacle, Terumo) was placed in the right common femoral artery. Under fluoroscopic guidance, the vessels of interest were selectively catheterized using a 5-French angled tapered catheter (Glidecath, Terumo, Tokyo, Japan) and digital subtraction angiography was performed with images of the intracranial circulation.

The angiographic run identifying radiographic vasospasm was selected as the pre-verapamil study. After identification of radiographic vasospasm by the neurointerventionalist, the vessel of interest (anterior cerebral artery, middle cerebral artery, internal carotid artery, or basilar artery) was selectively catheterized and the microcatheter was positioned immediately proximal to the region of radiographic vasospasm. Next, 5–10 mg of verapamil was given intra-arterially through the microcatheter over 10–15 min. This was repeated for each vascular territory with concern for clinical vasospasm and evidence of radiographic vasospasm. Following treatment, one final subsequent DSA was performed to evaluate treatment effect. The catheters and wires were then removed, and hemostasis at the groin puncture site was achieved using a vascular closure device or manual compression. Patients were then returned to the neurosurgical ICU for continued care. Imaging data was anonymized and transferred offline for analysis.

### Perfusion

Techniques that quantify perfusion statistics via bolus tracking are well established for CT and MR^16–19^. For this work, the perfusion parameters were measured using these techniques from software developed at our institution. This software was initially developed and validated for CT Perfusion by direct comparison to results of data returned from DAWN protocol CT perfusion^20^. The specific mathematics used to derive the perfusion statistics have been described in detail for both CT and MR imaging and the specifics are outside the scope of this paper.

Special considerations were taken for this work to transform techniques utilized for 3D perfusion calculations to the 2D DSA space. Each voxel of a study is represented by a signal-intensity curve that describes the signal resulting from contrast traversal. Measuring changes in signal intensity over time allows for the corresponding volume of traversing contrast to be quantified. This contrast response curve captures the perfusion properties of the tissue. These techniques of direct bolus tracking have recently been demonstrated for DSA, with no standard methodology accepted for calculating perfusion metrics from DSA^14,15^.

**Figure 1:**
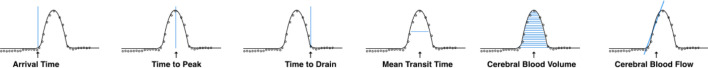
Graphical representation of individual intensity-time curves of contrast traversal across each voxel. Each chart demonstrates a single element of perfusion.

Cerebral blood volume (CBV) can be measured by comparing the amount of contrast at a specific point of interest (*x*, *y*) to the total amount of contrast administered from the arterial input. The contrast signal at any point can then be denoted as a function of time $C_{x,y}(t)$, and the specific contrast signal function at the arterial input is denoted as $C_{AIF}(t)$. The arrival time (*AT*) is defined as the time point when contrast first arrives at the voxel of interest. The time to peak (*TTP*) is defined as the time point where $C_{x,y}(t)$ is at maximum value. The time to drain (*TTD*) is defined as the time point where contrast has left the voxel of interest. The mean transit time (*MTT*) is defined as the amount of time between the two time points where $C_{x,y}(t)$ equals half of the the peak height. Figure 1 presents a graphical representation of the different perfusion metrics derived from direct bolus tracking over a single voxel. By directly measuring the *MTT*, cerebral blood flow (*CBF*) can be measured by the central volume theory with the set of equations below.

$$\begin{aligned} CBV_{x,y}= & {} \frac{\int ^\infty _{t=0}C_{x,y}(t)dt}{\int ^\infty _{t=0} C_{AIF}(t)dt}\\ CBF= & {} \frac{CBV}{MTT} \end{aligned}$$

### Perfusion analysis over vascular territories

**Figure 2:**
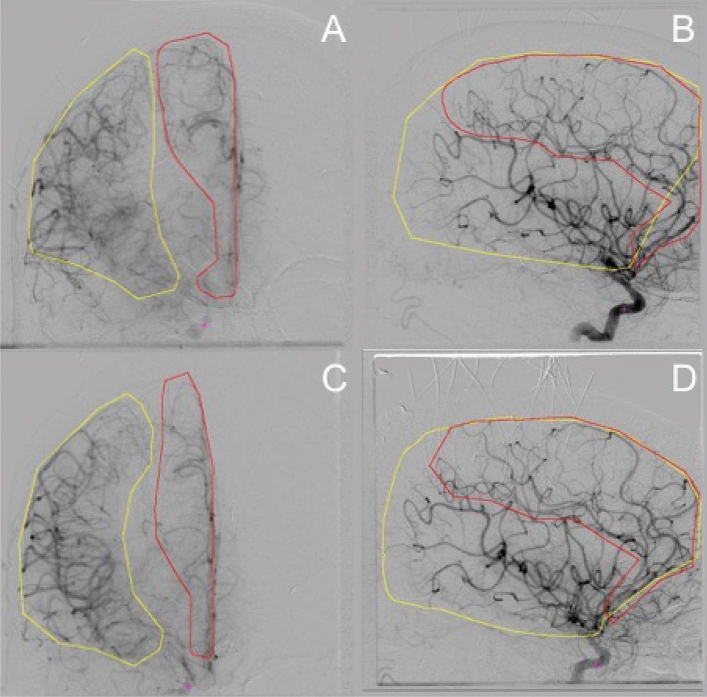
Demonstration of regions of interest (ROIs) over imaged hemispheres, analyzed for each study. In these examples, images (**A**,**B**) are from the pre-verapamil administration and images (**C**,**D**) are from the post-verapamil angiography. The yellow contours represent the MCA distribution and red contours represent the ACA distribution.

Changes in perfusion were compared between two DSA studies, one performed before, and the other after the administration of verapamil. In a retrospective fashion, angiographic cases were identified, anonymized, and then archived for review. Blinding to pre and post classification was performed. These studies were then exported to a workstation with MIM workstation installed (MIM Software, Cleveland Ohio). Vascular territories analyzed were defined to be the major vascular cerebral distributions (MCA/ACA/PCA/vertebrobasilar) and were manually defined for each study performed, to assist with reproducing the analysis. The vascular territories defined a region of interest (ROI), over which, perfusion statistics were calculated and presented as cumulative distributions for comparison (Fig. 2).

**Figure 3:**
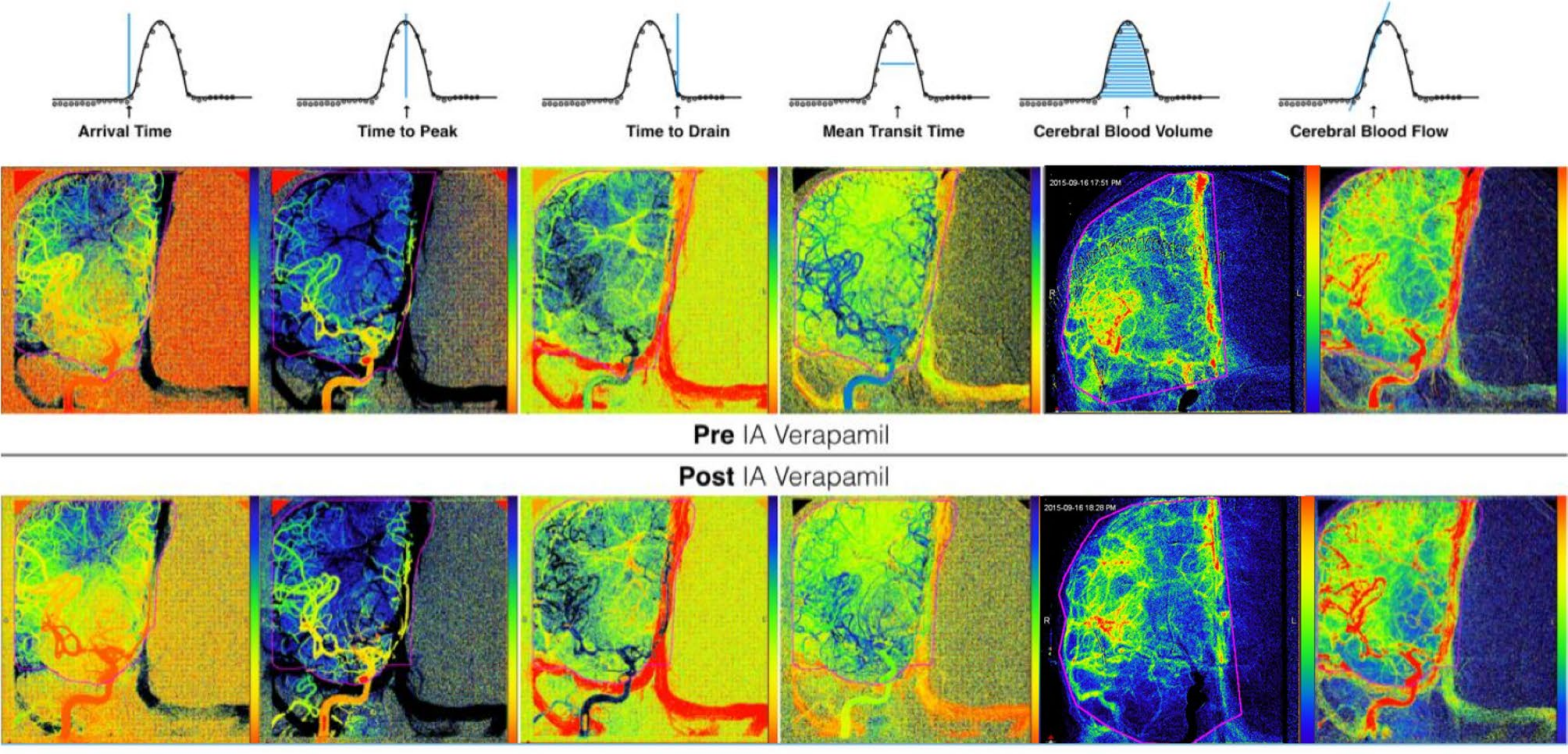
Demonstration of spatially resolved and colored representation of the different perfusion parameters. Here we are visualizing changes in each perfusion statistics for a single AP view from a pre/post verapamil administration.

For each voxel within the vascular ROI on each projection (AP/Lateral view), we measured and analysed the associated time-intensity curve, recording the AT, MTT, TTP, TTD, CBF and CBV. Each patient’s voxel-wise perfusion metrics were then combined and described as a distribution for the ROI for both the pre and post study. We selected a signal integration interval defined to be the initiation of signal enhancement and ending at the conclusion of the study or arrest of signal changes, whichever occurs earlier. This was performed automatically by our software. Because the software also performed a voxel-wise calculation of perfusion to produce a spatially resolved perfusion map, each voxel can be assigned a color corresponding to the relative perfusion statistics, scales normalized across the pre/post studies, therefore allowing perfusion maps to be provided for comparison (Fig. 3).

### Outcome measures

For each individual intervention we measured the changes in perfusion parameters over a vascular distribution demonstrating vasospasm in the pre-verapamil study compared to the post verapamil study. We specifically compared the arrival time, time to peak, mean transit time, and time to drain for the ROI. These values were directly compared to identify any statistical changes in the distributions by a comparison of means. Statistical significance was determined at $p<$ 0.05.

### Experimental design

This section describes the experimental protocol used in our study to evaluate the changes in cerebral perfusion between a pre-verapamil and post-verapamil angiogram. The hypothesis was that a statistically significant change in perfusion parameters exists when evaluating cerebral vasculature in vasospasm and comparing to the same vacsculature after treatment. The arterial input function (AIF) was defined to be the average dose-response curve values of a line spanning the width of the vessel from which the catheter was placed. It is possible to automatically segment this ROI, however, for this experiment, we wanted to ensure accuracy and elected to perform this manual segmentation by an expert. The vascular territories and AIF were outlined by a neurointerventional fellow to assist with aggregate statistical analysis. Aggregate distributions of each of the perfusion parameters were compared to measure changes across each perfusion metric. Perfusion analysis was performed on the pre-verapamil and post-verapamil angiogram sequences of each vascular territory treated for vasospasm. For these experiments we noted the dosage of verapamil infused for each territory, however, we did not include this as a factor for sub-analysis. If a patient required multiple IA interventions for persistent vasospasm, each treatment session was analysed independently.

## Results

Patient demographics are summarized in Table 1. Hunt-Hess scores were bimodally distributed with 2 and 4 being the grades with highest number of vasospasm cases. Fisher scores were 3 for twenty-one patients, and 4 for sixteen patients at presentation. Patients with supratentorial aneurysms represented a larger proportion of patients necessitating IA Verapamil (83.8%), one patient was determined to be angio-negative (3.2%), and the remainder were infratentorial aneurysms (13%). In terms of timing, the distribution of symptomatic vasospasm occurred in roughly a Gaussian distribution with vasospasm occurring on day 9.4 ± 4.2 days.

**Table 1:** Aggregate demographics of studied population.

	M	F	Total
Total	7	32	39
Average age	48.6	51.5	
**Hunt Hess grades**			
I	1	0	1
II	2	12	14
III	1	6	7
IV	3	10	13
V	0	3	3
**Fisher scale**			
III	4	17	21
IV	3	14	17
**Location of aneurysm**			
AComm	0	11	11
MCA	2	4	6
PICA	1	5	6
PComm	1	4	5
ACA	1	2	3
Basilar	0	2	2
Ant. choroid	1	0	1
Vertebral	0	1	1
ICA terminus	0	1	1
Angio. negative	1	0	1

39 patients were identified with bimodal distribution of Hunt-Hess grades, all patients were at least Fisher 3 or higher.

**Figure 4:**
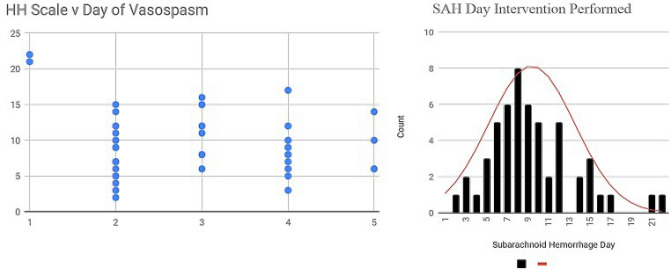
(Left) Scatter plot of days after presentation (y-axis) each patient (dot) underwent IA therapy for each Hunt Hess grade (x-axis). (Right) Cumulative histogram of day after rupture patients required IA therapy with Gaussian Distribution applied (red line).

Figure 4 demonstrates the relationship between Hunt-Hess scores relative to the day of vasospasm.

Of the patients identified, our perfusion angiography software was able to successfully process 82% of the cases (32 patients). Excluded cases included 4 patients whose imaging suffered from significant motion artifact either during the pre or post study, 2 patients were excluded because the pre/post images were of different views, and 1 was excluded for different magnifications of image capture during either the pre and post verapamil injections. In total, there were 95 doses of IA verapamil administrations performed within the successfuly processed patient population.

Changes to perfusion metrics were compared in aggregate by analyzing statistical features of the distribution over different vascular territories for the pre-administration and post-administration studies. For each perfusion parameter, we evaluated changes to the mean, median, standard deviation, kurtosis and skew between the pre and post ROI. Table 2 summarizes the clinically relevant average changes in perfusion parameters measured over each ROI after treatment. We found that statistically significant changes can be measured in the AP views, specifically decreases in the AT and TTP for both MCA/ACA distributions, and decrease in MTT for AP views of the ACA distribution.

**Figure 5:**
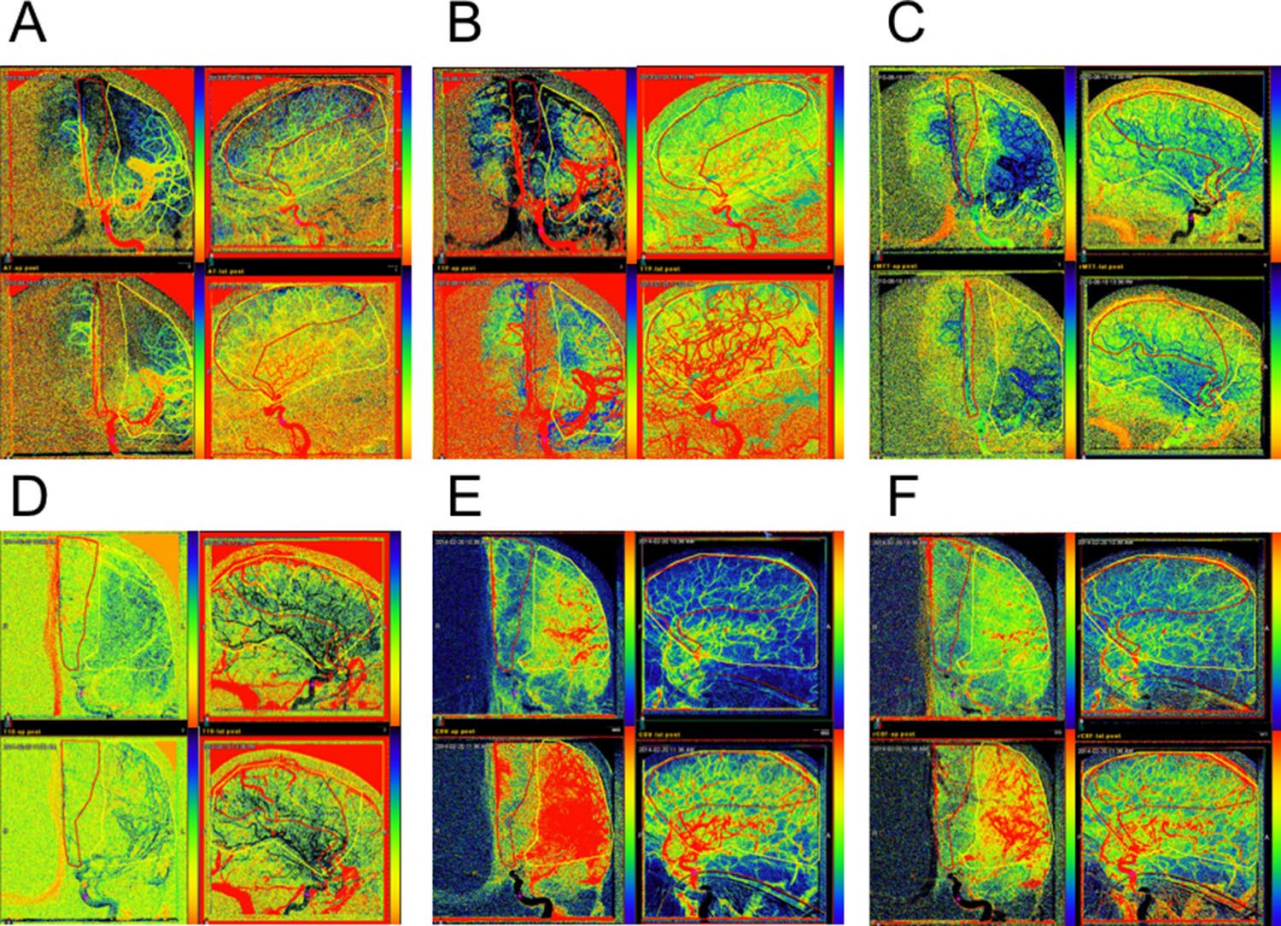
Graphical representation of perfusion parameters from a select patient illustrating changes in perfusion parameters after administration of verapamil. Each set of 4 images, observed clockwise from top-left represent the pre-AP, pre-LAT, post-LAT, and post-AP for a single perfusion parameter. (**A**) Arrival Time. (**B**) Time-to-Peak. (**C**) Mean-Transit Time. (**D**) Time-to-Drain. (**E**) Cerebral Blood Volume. (**F**) Cerebral Blood Flow. Clinicians are provided with these parameter maps to graphically visualize changes in AT, TTP, MTT, TTD, CBV, and CBF. To provide accurate comparisons between studies, each set is normalized and color coded over the same scale. By convention, red indicates increased perfusion $(\downarrow AT, \downarrow TTP, \downarrow TTD, \downarrow MTT, \uparrow CBF, \uparrow CBV)$. Black and blue indicate decreased perfusion $(\uparrow AT, \uparrow TTP, \uparrow TTD,\uparrow MTT, \downarrow CBV, \downarrow CBF)$.

When displaying the perfusion parameters individually, changes between the pre- and post-angiography become apparent (Fig. 5). The computation of perfusion parameters for a single patient took 0.7 s of computational time on a dual core laptop (MacBook Air 1.7 GHz Intel Dual Core i7 with 8 gb ram, Apple Cupertino, Ca). In principle, faster computation can be achieved if necessary, with parallelization.

**Table 2:** This table presents the mean change and 95% confidence interval for each perfusion statistic over each ROI for the pre/post verapamil dataset.

	${\Delta }$AT (s)	${\Delta }$TTP (s)	${\Delta }$TTD (s)	${\Delta }$MTT (s)
AP MCA	− 0.60 ± 0.48*	− 1.2 ± 1.1*	− 0.008 ± 0.3	− 0.52 ± 0.87
LAT MCA	− 0.30 ± 0.47	− 0.37 ± 1.1	0.17 ± 0.59	0.07 ± 0.91
AP ACA	− 0.66 ± 0.59*	− 1.6 ± 1.3*	− 0.0008 ± 0.35	− 1.1 ± 1.0*
LAT ACA	− 0.29 ± 0.52	− 0.31 ± 1.2	0.11 ± 0.64	0.07 ± 0.91
AP basilar	− 0.70 ± 2.0	0.45 ± 2.2	0.37 ± 0.79	-2.1 ± 7.0
LAT basilar	0.12 ± 1.6	1.4 ± 3.4	0.49 ± 0.87	2.5 ± 3.0

*Indicates statistical significance.

## Discussion

Perfusion analysis for DSA is not routinely performed nor is it standardized. Prior work has demonstrated initial applications of DSA perfusion analysis with respect to stroke and peripheral vascular disease^14,15^. With this work we present a methodology for real-time cerebral perfusion analysis to be performed on cerebral angiography for the evaluation of endovascular therapy for treatment of vasospasm following an aSAH. By examining the metrics of perfusion via bolus tracking, we are able to provide clinicians with real-time perfusion maps that graphically highlight hypoperfused tissue as well as changes in perfusion as therapeutic interventions are performed. Providing clinicians with temporal data, namely, changes in perfusion between runs, we envision this technology being used to assess treatment efficacy. One advantage over classic CT and MR perfusion is the high vascular resolution of DSA, the potential to selectively resolve smaller vascular territories, and its immediate use during interventional procedures.

Using perfusion maps, we retrospectively reviewed a series of patients to statistically analyse changes in perfusion metrics after IA verapamil used to treat cerebral vasospasm. We demonstrated statistically significant changes between pre- and post-IA perfusion metrics. Specifically, by evaluating the AP arrival time, time-to-peak, and mean-transit-time, we were able to discern statistically significant changes in both the ACA ($\Delta$AT, $\Delta$TTP, $\Delta$MTT) and MCA ($\Delta$AT, $\Delta$TTP) vascular territory parameters. Other groups have reported similar results using CT perfusion^21^. We were unable to identify statistically significant changes in the PCA or vertebrobasilar territories due to the low sample size of our dataset (n = 3). Furthermore, there is no control population or territories to compare these results to in our study. Future work may consider evaluating the non-treated vascular territories for a comparison.

In addition to providing clinicians with immediate verification of efficacy, this quantification can guide further studies to determine appropriate dose titration for IA therapies. We anticipate these techniques will be useful in understanding various underlying pathophysiologic pathways that contribute to vasospasm and delayed cerebral ischemia as perfusion deficits are directly observed and can be correlated to the biologic mechanisms of injury.

With regard to weaknesses, this retrospective study suffers from various limitations. The angiographic image capture protocol was not identical between our neuro-interventionalists. During the review of the 95 intra-arterial interventions performed, we identified cases where patients were spared radiation exposure by having a single internal carotid artery aquisition identifying A1/A2 and M1 stenosis, followed by super-selective catheterization to those territories, administration of verapamil, and then a post-administration internal carotid artery aquisition demonstrating improvement in vascular segment patency. This provided us with only one set of pre/post verapamil studies capturing multiple interventions.

Despite the excellent resolution of DSA, several shortcomings exist when using these studies to quantify tissue perfusion. Inherent to the technology, there exists noise resulting from the subtraction of a random distribution of X-Rays^22^. Another limitation is the motion of the patient during image acquisition creates both a temporal and spatial blur as image subtraction is not adequately aligned after subsequent frames. As DSA is a 2D projection, it is difficult to correct for any motion artifact arising from patient motion, resulting in errors of perfusion, which become significant when attempting to quantify perfusion over small vascular territories.

Finally, perfusion analysis performed on 2D projections of 3D vascular territories inherently results in aggregate perfusion of multiple territories. This limitation has been detailed and studied in other work^14,15^. Though this overlap is somewhat reduced by the selective arterial injections, the perfusion parameters differ slightly from their MRI and CT counterparts due to the lack of depth resolution. Our work does not deconstruct the dose response curve into the component curves of the multiple vascular territories that exist within each projection, however, this is a critical step for analysis in future work.

Despite these limitations, gross deficits of perfusion can often be seen on the original DSA, and quantitative changes can be observed over the parametric representation of perfusion statistics. Future work is still required to quantify the dose-dependant changes that verapamil infusion has on each vascular territory as well as the durability this therapy has when treating vasospasm. This will be the focus of our future work.

## Conclusion

With this work we have described how real-time perfusion analysis can provide an objective parameterization of DSA to assist physicians in creating care plans. Evaluating changes in direct bolus tracking dynamics of DSA, a voxel-by-voxel perfusion analysis provides a basis for quantitative analysis in real-time. Furthermore, this work demonstrates how we are able to display changes to perfusion across different studies for the same vascular territory to the provider, providing an evaluation of perfusion changes which can assist in determining the optimal therapeutic intervention. These maps provide very high spatial resolution (1024 × 1024) in our dataset. When reviewed side by side, each perfusion metric can be used to accentuate subtleties that are difficult to observe in a single dynamic series. We predict that these images can assist experts to better identify vascular territories which are hypoperfused, characterize temporal derangement in flow dynamics, as well as provide a direct quantification when assessing reliance of collateral flow.

Future work will focus on using this technology to titrate the appropriate dosage of IA verapamil and to quantify the durability of this treatment as measured by clinical correlations in a patient’s hospital course. Further, we will continue to refine our calculations in order to produce more statistically significant values for more perfusion metrics. Subsequently, studies will also focus on establishing the sensitivity and specificity of our technique by comparing against established perfusion studies. Once these are established, the true clinical utility of the software can be assessed in a prospective fashion.
